# Getting It Out There: Reflections on the Process and Impact of Public Engagement Activities in a Study on End‐of‐Life Care Planning With People With Intellectual Disabilities

**DOI:** 10.1111/hex.70653

**Published:** 2026-03-26

**Authors:** Andrea Bruun, Amanda Cresswell, David Jeffrey, Leon Jordan, Richard Keagan‐Bull, Jo Giles, Sarah Swindells, Meg Wilding, Nicola Payne, Gemma Allen, Rhidian Hughes, Elizabeth Tilley, Sarah L. Gibson, Rebecca Anderson‐Kittow, Irene Tuffrey‐Wijne

**Affiliations:** ^1^ Department of Public Health, Children's, Learning Disability and Mental Health Nursing Kingston University London London UK; ^2^ Dimensions Reading UK; ^3^ MacIntyre Milton Keynes UK; ^4^ The Mary Steven's Hospice Stourbridge UK; ^5^ Voluntary Organisations Disability Group London UK; ^6^ Open University Milton Keynes UK

**Keywords:** end‐of‐life care planning, engagement, intellectual disability, patient and public involvement

## Abstract

**Introduction:**

Many tools and interventions developed from research projects are not properly implemented in health and social care practice. Engaging with stakeholders is an important part of the implementation process when developing, sharing and embedding new tools. The Victoria & Stuart Project was a 2‐year project that co‐designed a toolkit of resources and approaches to support end‐of‐life care planning for people with intellectual disabilities. It had an inclusively delivered dissemination and engagement workstream to raise awareness of the toolkit throughout the project. In this paper, we describe and reflect on the project's public ‘inspiring and informing’ engagement activities.

**Methods:**

Engagement activities included a project website, weekly blog and newsletter. The project held webinars and was promoted on various social media platforms. One‐to‐one stakeholder consultations with all members of the project's Research Advisory Group were held. Presentations were given to both academic and non‐academic audiences.

**Results:**

Engagement activities were labour‐intensive and time‐consuming, particularly when creating regular content such as weekly blogs and social media posts. It became obvious that team members did not have expertise in marketing or communication skills and had to learn from experience. In addition, it was difficult to build and maintain a following with a changing social media landscape. There is evidence that the project is on its way to meet the project's engagement‐related medium‐term outcomes, such as being included in national palliative care reports and accreditation programmes as well as international guidelines.

**Conclusion:**

Public engagement is a complex, uncertain and non‐linear undertaking, requiring continual reflection and refinement in response to changing circumstances within and beyond the research. We believe a key driver for successful engagement has been employing researchers with intellectual disabilities as well as working actively with core stakeholders throughout the project. Our engagement recommendations are: (1) Involve people with lived experience; (2) Plan and allocate time; (3) Include social media and/or marketing roles in research; (4) Tailor to the audience and platform; (5) Make it engaging and fun; (6) Learn how to make videos; (7) Keep presenting your work; and (8) Make time for stakeholder consultations.

**Patient or Public Contribution:**

The research team included four researchers with intellectual disabilities (A.C., D.J., L.J. and R.K.‐B.). Researchers with intellectual disabilities have been part of every step of the research process; from study design to data collection and analysis to dissemination of study findings, including engagement activities. Palliative care and intellectual disability service provider representatives (G.A., M.W., N.P., R.H. and S.S.) have been closely involved throughout the project. The research was supported by a Research Advisory Group comprising a variety of stakeholders, including people with intellectual disabilities, families, representatives from intellectual disability and palliative care organisations, and policymakers.

## 1 Introduction

We are a team of researchers who conducted a 2‐year project developing an end‐of‐life care planning toolkit for people with intellectual disabilities, aimed at enabling their involvement in decisions around their own care and support at the end‐of‐life [1]. Patient and public involvement and engagement (PPIE) was at the heart of the project [2], where people with intellectual disabilities, family carers, and health and social care staff were actively involved as researchers, advisors and co‐production partners. This paper is focused on the engagement aspect of PPIE, related to when, where, and how information about the project was provided and disseminated to the public [2]. In this context, our various ‘publics’ were service managers, staff and carers who would be involved in facilitating the uptake of the toolkit in health and social care practice. In this paper, we reflect on the rationale, implementation, and impact of our engagement activities with these different stakeholder groups.

Many interventions developed and found to be effective in health services research studies fail to translate into meaningful patient care outcomes across multiple contexts [3]. There are important factors to consider when implementing interventions within health and social care services. Major barriers to implementation include organisational culture; lack of time, skills and knowledge among practitioners; and access to research evidence and information [4]. Availability, user‐friendliness, and accessibility of an intervention have been found to be enabling factors for its implementation within intellectual disability care, as well as management support and input [5]. As a response to the lack of sustained implementation of interventions, the consolidated framework for implementation research (CFIR) was developed. CFIR is a framework that promotes implementation theory development and verification about what works where and why across multiple contexts [3].

Before coming to the stage of embedding and then evaluating the implementation of an intervention, the service needs to know about it. This requires researchers to effectively engage with services and their staff to make them aware of an intervention and to persuade them of its potential utility and effectiveness. Of relevance here from the CFIR is the statement: ‘Attracting and involving appropriate individuals in the implementation and use of the intervention through a combined strategy of social marketing, education, role modelling, training, and other similar activities’ [3], p. 11. Voss et al. also identified announcing and stimulating the use of the innovation as a facilitator for its uptake in their review of palliative care innovations in organisations providing care for people with intellectual disabilities [6]. This involved various dissemination strategies and materials used to promote implementation, which included distributing flyers, organising meetings, mentioning the innovation in newsletters, and making use of social media [6]. The review by Collingridge Moore et al. [7] showed how raising awareness of the intervention to stakeholders outside those delivering it allowed for wider investment in improving palliative care in terms of time and resources in long‐term care facilities. Engaging with a variety of stakeholder groups facilitate the implementation of a palliative care intervention within health and social care services.

Engagement activities should be adapted and tailored to each stakeholder group. Most research‐based literature is not easily available or readily understood by practitioners [8], which forces researchers to pursue other ways of engaging with stakeholders. The format and language matters, and it is important for researchers to consider who they are engaging with, and whether they are speaking to a policymaker, a commissioner, a health and social care professional, or a patient or carer. If stakeholders include people with an intellectual disability, there are particular challenges with understanding complex language and abstract concepts involved in research, and easy‐read materials and videos may be needed [9]. Staff within adult social care services in England, over half of whom have no relevant social care qualifications [10], may benefit from plain English communications. Sustained relationships between researchers and practitioners will improve implementation of evidence‐based innovations, as does access to usable and applicable evidence, and the inclusion of the ‘user voice’ [11].

## 2 Aim

In our research, we recognised the criticality of effective engagement activities in supporting a pathway towards long‐term toolkit implementation and ultimately improved patient‐centred outcomes. This paper explores some of the tensions inherent in the sphere of public engagement for research, reflecting on the rationale, implementation, and impact of activities undertaken for The Victoria & Stuart Project (see [1]).

The objectives of this paper are to:

1. Present and describe a range of the project's engagement activities.
2. Identify the challenges, benefits and impacts of these activities.
3. Develop public engagement recommendations for future research projects.

## 3 Materials and Methods

### 3.1 The Victoria & Stuart Project

The Victoria & Stuart Project was a 2‐year research project focused on co‐designing a toolkit of approaches and resources for end‐of‐life care planning with people with intellectual disabilities in social care services [1]. The project involved two universities (Kingston University London and The Open University), two intellectual disability service providers (MacIntyre and Dimensions), one umbrella disability organisation (The Voluntary Organisations Disability Group), and a hospice (The Mary Stevens Hospice) in the United Kingdom. The research team comprised researchers with and without intellectual disabilities. The project was supported by a Research Advisory Group of self‐advocates, family members as well as intellectual disability and palliative care organisations and policy makers. The final output of the project was an end‐of‐life care planning toolkit available on the study website (www.victoriaandstuart.com). The project commenced in April 2022 and concluded in July 2024, following a 3‐month no‐cost extension. The toolkit was launched at the end of June 2024.

#### 3.1.1 Engagement Objectives

Within the project, we established a workstream dedicated to dissemination and stakeholder engagement. The rationale for embedding significant stakeholder engagement from the start of the project, well before the toolkit was developed, was to take account of the time required for effective awareness‐raising and the expectation that this would increase the likelihood that wider stakeholder groups were ready to engage with and use the resources once developed.

The main objectives for our engagement activities were to meet the following medium‐term project outcomes (1–5 years post‐project): (1) end‐of‐life care planning resources are widely known and used; (2) resources are easily accessible to the health and social care workforce; and (3) health and social care services and staff understand their role in end‐of‐life care planning for people with intellectual disabilities, and can make use of available resources.

### 3.2 Methods

This paper describes and reflects upon public engagement activities in research. It does not report on a formal evaluation of these activities, and for this reason it does not include details of participants, data collection nor analysis.

Instead, we report on specific project engagement activities undertaken between April 2022 until March 2025. We have used the Ten Principles of High Quality Engagement [12] to guide our reflections. The National Co‐ordinating Centre for Public Engagement (NCCPE) describes three broad forms of engagement activities: consulting; collaborating; and inspiring and informing. In this paper, we focus on the latter. We follow the Guidance for Reporting Involvement of Patients and the Public (GRIPP2) checklist for reporting PPIE in research [13]. We discuss what we were able to successfully activate in terms of public engagement (and how), as well as the challenges that arose (and how we strove to navigate these).

Our reporting on the perceived impact of our engagement activities is informed by qualitative data collected via social media posts and email correspondences, conversations, comments, and questions during conferences, webinars and other events, and our own personal reflections. Metrics are also captured for the website, blogs, newsletters, and webinars to indicate levels of engagement.

#### 3.2.1 Stakeholders

The end‐of‐life care planning toolkit was developed specifically to be used by staff within adult intellectual disability settings with the people they support. Whilst the toolkit resources needed to work well for people with an intellectual disability, and were co‐developed with them, they were not the main target of our engagement activities, as they were unlikely to access or use the resources without support. Therefore, the main ‘public’ were managers and direct support staff within social care services. However, stakeholders also included families and healthcare professionals, especially those within hospital, primary care, and palliative care settings, as their awareness of the toolkit might facilitate implementation. Other stakeholders were commissioners and those producing national guidance, standards or training, such as the Care Quality Commission (England's independent health and social care regulator and inspector [14]), NHS England and Hospice UK. All these stakeholder groups were represented on the project's Research Advisory Group.

#### 3.2.2 The Project Team

The engagement activities described below were initiated and carried out by the researchers at Kingston University London who led the project, consisting of a professor (I.T.‐W.), two postdoctoral researchers (A.B. and R.A.‐K.), one postgraduate researcher (S.G.), and four researchers with an intellectual disability (A.C., D.J., L.J., and R.K.‐B.), and their support research assistant (J.G.). All worked part‐time. They brought in members of the wider research team, members of the co‐design group (see [15]), and Research Advisory Group members to help produce and/or disseminate content. An important part of the engagement strategy was the prominent involvement and inclusion of people with an intellectual disability.

### 3.3 Engagement Activities

#### 3.3.1 Project Website

A university‐external, separate domain was purchased, and the team worked together with a website developer to build a project website. Members of the research team learned how to manage the website, and it was regularly updated throughout the project. The website included basic information about the project, including accessible easy‐read project information, and team members. It had a video tab (e.g., conference and toolkit resource demonstration videos). Webinar recordings were uploaded and announced in a separate webinar tab. People could request to receive the project newsletter and blog via email through a ‘sign‐up’ button. Some study results were shared on the website before they were published in peer‐reviewed journals such as findings from the scoping review (see [16]), where a shortlist of promising end‐of‐life resources were made publicly available.

#### 3.3.2 Project Blog and Newsletter

A strategic communications plan was put in place in January 2023. This involved a weekly blog written by a diverse group of people involved with the project, including researchers with and without intellectual disabilities, co‐applicants from intellectual disability or palliative care services, and members of the co‐design group (see [15]). The blog targeted different types of audiences through diverse formats such as videos and podcasts and content such as reflection pieces, project activity descriptions (e.g., focus groups, conferences, and co‐design workshops), and discussions of relevant academic literature. The blog launch involved creating and sharing short introduction videos of 15 project team members on social media. The purpose of the blog was to increase visibility and engagement for the study, whilst it was anticipated that guest editorials from different members of the project team would enable a wider reach across social media platforms and networks. For example, blog posts written by someone with an intellectual disability who received support from a major national service provider, would be promoted within that service. The blog thus included multiple perspectives as well as breaking down content into a more accessible format for non‐academic audiences.

As part of the communications plan a monthly newsletter was set up. The e‐mail newsletter included information on project progress, relevant events, and blog entries from the past month. It was sent out through the website and was kept short and engaging with pictures.

#### 3.3.3 Webinars

The project involved webinars for each of the project's workstream. These were held in collaboration with The Palliative Care for People with Learning Disabilities (PCPLD) Network, who were experienced in hosting webinars and have a large pre‐existing audience. The webinars were shared with the organisation's emailing list as well as on their website. Webinars were co‐chaired by the project lead and researchers with an intellectual disability. Webinar content was presented inclusively and involved videos to engage a wide audience. The webinars were recorded, uploaded to The PCPLD Network's YouTube channel, and subsequently shared on The Victoria & Stuart Project website.

#### 3.3.4 Social Media Platforms

The project had a Twitter account where blog entries, newsletters, and webinars were posted. Progress posts were also created to increase engagement, for example, by sharing an activity from a workday in the office. Papers were shared as well as pictures and posts from conferences or other presentations. The Twitter platform was chosen as it allowed for engagement with a wide range of stakeholders, including both academic and non‐academic audiences. The project also had a YouTube channel where videos were posted.

#### 3.3.5 Individual Stakeholder Consultations

The project's Research Advisory Group included 25 members who met five times throughout the project. They reviewed, evaluated, and discussed project progress and findings. At the last meeting, there was a low attendance rate, which provided the team with limited feedback regarding toolkit dissemination. It also became evident that the research team could benefit from more in‐depth conversations with each member to gain insights into relevant links and networks for dissemination. This led the research team to have one‐to‐one meetings with each advisory group member to ensure impact and wider spread of the final toolkit. Some members were representatives from the same organisations and had their meeting together.

#### 3.3.6 Presentations and Conferences

Presentations for different stakeholder groups were held, including academic and non‐academic audiences. Most non‐academic presentations involved different health and social care staff audiences rather than people with intellectual disabilities themselves. This is due to staff being the ones using the toolkit in practice, and for whom the toolkit was developed, as outlined previously. We presented to staff from the Care Quality Commission, GPs, local intellectual disability groups and networks, and palliative care social workers. Most of these were invited presentations. We also had conference stalls to display our resources, for example, at Learning Disability England's conference. Presentations were held by researchers with and without intellectual disabilities. They were tailored to each stakeholder group and kept engaging and fun with videos and pictures, often with limited focus on academic aspects such as theory and methods. A video version of the final project report for the funder was also created.

## 4 Results

In this section, we present our reflections on and discussion of impact of the engagement activities described previously, and then we share the challenges of undertaking these.

### 4.1 Reflections on and Discussion of Impact

#### 4.1.1 Engagement Activities and Feedback

A summary of the outputs of the activities is presented in Table 1.

**Table 1:** Engagement activities.

Activity	Numbers	Metrics	Time period
Website activity		29,064 Page views	April 2022–July 2024
		12,026 Site Sessions^a^	
		7269 Unique visitors^b^	
Blogs	68	4919 Views	January 2023–July 2024
		2271 Unique visitors	
		4 Comments	
		30 Shares	
Newsletters	6	35 Unique opens^c^	January–December 2023
		13 Unique clicks^d^	
Webinars	6	4687 views^e^	May 2022–June 2024
		797 attendees^f^	
Stakeholder consultations	19 online meetings with 24 members		January–April 2024
Presentations	38		April 2022–March 2025

^a^ A session is a visit to the website site. It ends after 30 min of inactivity.

^b^ The number of people who visited the website site. A visitor is considered unique when they connect from a different browser or device (IP address).

^c^ The number of unique contacts that opened the newsletter email.

^d^ The number of recipients who clicked a link in the email (except for unsubscribes).

^e^ Webinar views captured on 27 March 2025.

^f^ Attendee numbers were noted for four out of the six webinars.

It is difficult to assess whether the engagement activities have been successful in meeting our medium‐term project objectives. Whilst the metrics in Table 1 indicate that we have gone some way in meeting the objective that the toolkit is ‘widely known’, this cannot establish to what extent the toolkit is ‘used’. It is particularly difficult to know which engagement activities have been most influential or crucial. There is some evidence that the activities have had a positive impact, including qualitative feedback received via email, webinar responses, and conference evaluations. For example:

This has quickly become a vital resource for us, helping everyone think about and plan for end‐of‐life care. We use the toolkit in workshops with people with intellectual disabilities and are rolling this out throughout our region. [Lead nurse, intellectual disability service provider; received via email].

#### 4.1.2 Follow‐Up Projects

After the project, several international research groups contacted the team to enquire about translating toolkit resources. This led the team to collaborate with Swiss palliative care researchers from the PAL‐LINK Project [17] on translating resources, following co‐design approaches. The Swiss team first came across the project during an online search and then attended several of the project presentations. The Swiss team reported ‘The card sets are eagerly awaited here in Switzerland (and will surely soon be known in Germany and Austria too)’, when the German cards were published online in October 2025. This collaboration has not only led to a new translated resource but also provided key learning points on how to translate such resources in the future. A paper on this project is currently under review.

Another example is that after a small workshop for Marie Curie UK, a major palliative care organisation in the United Kingdom, funding was sought and awarded for a collaborative project on distributing printed versions of the toolkit free of charge [18]. The project came about as an initiative from a workshop participant after having heard that printing was a key barrier to toolkit uptake and use in social care practice (see [19]). From 1 September 2025, 1000 free printed copies of the toolkit resources were made available to order. By 23 September, all initial stock had been ordered. Findings from this project has led the organisation to consider and explore the opportunity for continuing this service in the future as well as linking to the project on their ‘palliative and end of life care for people with a learning disability’ webpages for health and social care professionals [20]. This is a lesson learned on how engagement through a small workshop with 18 people can lead to significant impact in terms of sharing a new resource with a large number of practitioners and services.

The team is regularly approached by people who have seen or heard a presentation at events and conferences. For example:

I am a learning disability nurse working in [UK city] and have an interest in improving advanced care planning for people with learning disabilities. I have enjoyed listening to the webinars and following the project. The toolkit looks really helpful and I will be spreading the word! [Intellectual disability nurse; received via website contact form].

We also receive regular enquiries about using, altering, or incorporating parts of the toolkit resources from other researchers and professionals as well as being invited to speak at events and starting to be mentioned by palliative care or funeral services [21] and other projects on this topic such as the West Yorkshire ICB‐funded ‘Talking About Dying’ project [22].

The project is mentioned in national end‐of‐life care guidelines [23] and accreditation scheme training such as the Gold Standard Framework. In 2025, the project was listed in internal WHO guidelines on ‘Good practices on disability‐inclusive health’ [24]. The project also won two PPIE awards in 2025: The Marie Curie Tammy Prescott Patient and Public Involvement Award and The Association for the Study of Death and Society Public Engagement Award. One of the panels stated:

The panel felt that the team showed a strong commitment to involving patients and the public throughout the research… Overall, the judging panel felt that their involvement was at the heart of this research. Their meaningful impact is clear from the toolkits which are now publicly available [Marie Curie Tammy Prescott Patient and Public Involvement Award 2025 panel feedback].

#### 4.1.3 Impact of Involving People With Lived Experience in Engagement Activities

We do believe one of the main reasons for the positive impact detailed above has been the involvement of people with lived experience and the co‐produced nature of the research. We worked very closely with core stakeholders and drafted them in to help communicate our messages and asked them to help us thinking about what those messages actually are. This was important not only with regards to people with intellectual disabilities, but also other target groups, such as social care staff. For example, we asked families, intellectual disability support workers, and service managers to review our messages and made videos including their voices and perspectives. Having almost 200 people, including 36 researchers and people with an intellectual disability, actively involved in the project has been crucial in delivering effective engagement activities.

#### 4.1.4 ‘Make It Fun’

The investment from the team in involving people directly or indirectly by producing accessible videos with a strong emphasis on the voices of people with intellectual disabilities aided our engagement as it added credibility to our work and displayed co‐production and our inclusive values that resonated with stakeholders. During the co‐production process, we received a strong message from people with intellectual disabilities that they wanted discussions about end‐of‐life care to be ‘fun’, despite (or perhaps because of) the difficult topic. We used music, dance, and games to make messages accessible. As they were involved in creating video outputs, which we used widely in webinars and presentations, this sense of accessibility and fun was also communicated to other audiences. It seemed particularly effective, as we have received consistent feedback that the videos were memorable, moving, and inspiring.

### 4.2 Reflections on Challenges

#### 4.2.1 Time

A key challenge was the time commitment needed for the engagement activities. In other sectors, people are employed in specific roles to oversee and create social media and marketing strategies. The project's social media presence and general activity needed to be maintained, which meant that regular content was needed. This added to the work burden and was a significant time commitment not budgeted for in the project.

Creating weekly blogs was a lot of work and required planning and time. Encouraging team members to frequently write blog content whilst also having many work commitments and conflicting priorities was challenging. This was despite having a big team who could contribute. The social care collaborators were working in a finance and resource‐stretched sector. Researchers with an intellectual disability needed support from other team members to write content, which was available, but their limited work hours meant that their time was precious and had to be prioritised and spent on other important research aspects. Similarly, a lot of the team members were only working part‐time on the project with limited time for blog writing. This meant that many blog entries were written by the same team members. Some content also had to be very simple, for example just sharing a conference poster, due to lack of other content.

#### 4.2.2 Expertise

People in social media or marketing roles have expertise in doing this work, which traditional academics lack. None of the team members were trained or educated in website development, social media content, or video‐making. They were self‐taught and learnt such things ‘on the go’, which was also time‐consuming. The team was lucky to have access to video editing software and easy‐read symbols through university access or paid subscriptions. However, better quality content could have been created had the right expertise and knowledge been available.

#### 4.2.3 Navigating the Social Media Landscape

It takes time to build up engagement and get a regular audience on social media. Time is needed to find the right and relevant people to follow on social media, who may share your content and help with spreading the word. Maintaining followers is time‐consuming, and they can be quickly lost as well. At the end of the project, Twitter became X, which changed the platform, and—as part of a wider global trend and socio‐political issues (e.g., X became a place that no longer felt safe for many people)—many users left, lowering overall engagement and uptake [25]. The change also meant that engagement metrics around account posts and followers could no longer be obtained for free, following a long period of limited reporting of this. A similar platform with the same stakeholder reach has not yet been established.

#### 4.2.4 Newsletters

The plan was to have a monthly newsletter. After the first couple of newsletters, the team had to lower the frequency as there was not enough content for a monthly one. In research, things come in waves, where one month we would have plenty of things to share, and then the next one would be a quieter, less ‘newsworthy’, time of data analysis or ethics applications. As evident in Table 1, the newsletter uptake was also quite low. Much of the newsletter content was already covered in the weekly blogs, which made the newsletter redundant. After a year of only having sent out six newsletters, the team decided to end distributing them.

#### 4.2.5 Presentations

Giving presentations can also be time‐consuming. The team received regular invitations to present at different events, workshops, or meetings relevant to end‐of‐life care planning with people with intellectual disabilities. The team had to make a judgement about which presentations should be prioritised. This unfortunately meant that smaller local invites had to be turned down due to time constraints. However, the team was mindful that greater numbers do not always mean greater impact; sometimes five people in the room is enough and can create a ripple effect.

#### 4.2.6 The Need to Consider Audiences

In hindsight, we should have carefully considered our audience and stakeholders for every single engagement activity. It is important to consider who the audience is and create tailored content to them. Despite having reached and engaged with both people with an intellectual disability, their families, professionals, and policy makers, we probably spread ourselves too ‘thinly’. By honing in on what they would be interested in knowing, what their worries and needs were, we could have created much better tailored content. The needs of the person with an intellectual disability are very different from the needs of a policymaker. Although we are talking about the same topic, end‐of‐life care planning with people with an intellectual disability, each stakeholder is invested differently. We learned more about this during the one‐to‐one consultations with out advisory group members, and always had that in mind when giving presentations, but we should have incorporated this more strategically into our other activities, particularly our videos.

## 5 Discussion

PPI hugely influenced The Victoria & Stuart Project, and we believe this was essential to the positive impact the engagement activities and overall study has had, including multiple awards and both national and internal recognition. Involvement has been crucial for the project, especially the researchers, advisors, and co‐design members with intellectual disabilities, and it helped us emphasising the rationale, strong purpose, and context of the study, as noted by the NCCPE. However, writing this paper has also made us conscious of the essential nature and complexities of engagement.

The Research Excellence Framework (REF) is the UK's system for assessing the excellence of research in UK higher education providers. REF outcomes are used to inform the allocation of around £2 billion per year of public funding for universities’ research [26]. The REF includes an assessment of research impact, accounting for 20% of the funding allocation [27]. The pressure on researchers to demonstrate impact is thus increasing, with several tools and frameworks being developed to assess impact as well. Different approaches to assessing research impact make different assumptions about the nature of research knowledge, the purpose of research, the definition of research quality, the role of values in research and its implementation, the mechanisms by which impact is achieved as well as the implications for how impact is measured [28]. It has been argued that short‐term proximate impacts are easier to attribute, whereas benefits from complementary assets (e.g., development of research infrastructure, political support, and key partnerships) may accumulate in the longer term but are more difficult to fully capture [28]. As evident in our reflections on and discussions of impact, it is difficult to measure which of the engagement activities have been most successful and crucial to the project. We have to rely on our anecdotal evidence, as presented in the results section, in these cases.

Issues with the time commitment in relation to research engagement activities have been echoed elsewhere. Staff have mentioned time and resources as barriers to public engagement activities, and moreover that such activities are less valued compared to others such as teaching and publications [29]. Academics have also expressed that the pursuit of engagement may spread researcher capacity too thinly, taking time away from research and forcing researchers to look beyond their core expertise, prioritising breadth over depth [30]. The fact that our team members went the extra mile in terms of engagement is great, but we question whether it is sustainable or cost‐effective. There is a need for more professional communications professionals who have the right training and expertise [31].

This paper has highlighted that we need to be more aware and explicit, from project start, about the purpose and context of each engagement activity and its associated resources, costs, and efforts. However, the complex nature of engagement means that it is uncertain which activity may lead to greater impact, as the case with the small Marie Curie workshop leading to an unexpected follow‐up impact study. Perhaps reflecting and mapping potential impact outcomes related to activities as well as their associated costs would have been useful. The NCCPE recommends that evaluation is used strategically, and that work is reflected upon. We could have been better at doing this, trying to identify together the good ingredients of our engagement activities earlier and continuously. This paper has made us reflect on this and led to our recommendations presented in the conclusion.

Writing this paper made us aware of the complexity of the implementation, engagement, and impact research field and our own limited knowledge, experience, and proper training. It has been highlighted that there is a need for academics to receive training in the terms and concepts associated with the engagement agenda and to be informed about the need for both demonstrating reach and significance and actively look to find evidence for both in their interaction [32]. It would have been helpful to have included an implementation scientist in the research team. However, there was no room for this in the research budget. The NCCPE recommends that help is sought where needed and expert administrative support is available as well as working with those with relevant knowledge such as public engagement professionals. Such roles and work need to be budgeted for in future funding applications to ensure resources are in place.

Externally funded research projects also face challenges with what happens following project completion. Who continues to manage the project website, spread the word about the resources, and keep the project alive to ensure sustained impact is achieved? This is partly why these engagement activities were carried out throughout the project as there would be no time after the project when researchers would have other priorities. Externally funded project team members might be forced to leave the institution when their contract ends, and they have to find work elsewhere. Ideally, follow‐up funding would secure continuation of the project, but that is not always possible in a competitive research environment with low success rates. This leaves the responsibility with more senior members with permanent contracts. However, any work would be from goodwill and, again, someone going the extra (non‐sustainable and non‐cost‐effective) mile. We were unusually lucky that most of the team were able to continue this work by being successful in securing another grant that kept people employed at the university as well as having a supporting university behind us.

## 6 Conclusion and Recommendations

Public engagement is a complex, uncertain and non‐linear undertaking, requiring continual reflection and refinement in response to changing circumstances within and beyond the research. This paper has detailed impact and engagement activities for The Victoria & Stuart Project; a study co‐developing an end‐of‐life care planning toolkit with people with intellectual disabilities,the value of reflecting on the public engagement aspect of PPIE and used existing frameworks to do this. Writing this paper, using a systematic reporting guideline, has helped us to clarify what worked well and what we need to do better in future projects. Based on our experiences, we have developed the following recommendations:

1. **Involve people with lived experience**—as much as possible in engagement activities, they are what people remember the most, they touch people's hearts and often best at communicating key messages.
2. **Plan and allocate time**. Set aside time for your engagement activities. Plan the activities, particularly blogs and social media content, well in advance.
3. **Include social media and/or marketing roles in research**. Cost in marketing and communications staff into your budget. If that is not possible, then consider alternatives such as training for your team instead.
4. **Tailor to the audience and platform**. Think about who you are trying to engage with and what their worries, (accessibility) needs, and passions are, and then tailor content accordingly. Also think about which medium and platform to use to engage and reach them as well as tailoring content to the platform.
5. **Make it engaging and fun**—something that people will remember you for, do not be another ‘boring’ academic. Videos work well. Play to people's strength, if you have a poet, write a poem, if you have an artist, create artwork.
6. **Learn how to make videos**. It is an inclusive and engaging format and can be used in many different dissemination formats or find someone who can. It makes a difference.
7. **Keep presenting your work**—to a wide audience; reconsider whether a local smaller presentation is ‘not worth it’, it may open new avenues, make sure you do not always turn them down. Seek the opportunities yourself—cold canvas style.
8. **Make time for stakeholder consultations**. Discuss with key individual stakeholders how the project is relevant to them and how to move it forward. Think about key pivotal points during the project when this would be most useful. It also maintains a stronger relationship, and everyone has a chance to be heard and seen.

## Author Contributions

**Andrea Bruun:** investigation, writing – original draft, writing – review and editing. **Amanda Cresswell:** investigation, writing – review and editing. **David Jeffrey:** investigation, writing – review and editing. **Leon Jordan:** investigation, writing – review and editing,. **Richard Keagan‐Bull:** investigation, writing – review and editing. **Jo Giles:** investigation, supervision, writing – review and editing. **Sarah Swindells:** funding acquisition, conceptualisation, investigation, writing – review and editing. **Meg Wilding:** investigation, writing – review and editing. **Nicola Payne:** funding acquisition, conceptualisation, investigation, writing – review and editing. **Gemma Allen:** funding acquisition, conceptualisation, investigation, writing – review and editing. **Rhidian Hughes:** funding acquisition, conceptualisation, investigation, writing – review and editing. **Elizabeth Tilley:** funding acquisition, conceptualisation, investigation, writing – review and editing. **Sarah L. Gibson:** investigation, writing – review and editing. **Rebecca Anderson‐Kittow:** funding acquisition, conceptualisation, investigation, writing – review and editing. **Irene Tuffrey‐Wijne:** funding acquisition, conceptualisation, investigation, writing – review and editing.

## Ethics Statement

The paper does not involve participant data, thus ethical approval has not been obtained.

## Consent

The paper does not involve participants thus participant consent has not been obtained.

## Conflicts of Interest

The authors declare no conflicts of interest.

## Data Availability

Data sharing is not applicable to this article as no datasets were generated or analysed during the current study.
